# When to sample in an inaccessible landscape: a case study with carabids from the Allgäu (northern Alps) (Coleoptera, Carabidae)

**DOI:** 10.3897/zookeys.100.1531

**Published:** 2011-05-20

**Authors:** Ingmar Harry, Claudia Drees, Hubert Höfer, Thorsten Assmann

**Affiliations:** 1 ABL, Nägeleseestraße 8, D-79102 Freiburg, Germany; 2 Tel Aviv University, George S. Wise Faculty of Life Sciences, Department of Zoology, The National Collections of Natural History, Tel Aviv 69978, Israel; 3 Natural History Museum Karlsruhe (SMNK), Erbprinzenstraße 13, D-76133 Karlsruhe, Germany; 4 Institute of Ecology and Environmental Chemistry, Leuphana University Lüneburg, D-21335 Lüneburg, Germany

**Keywords:** Carabidae, mountain ecosystems, phenology, sampling effort, pitfall traps

## Abstract

While pitfall trapping is generally accepted as the standard method for sampling carabid beetles, this method has rarely been used in mountain ecosystems, mainly due to the high labour intensity it involves. As part of a research project in the German Alps, we investigated the phenologic appearance of adult carabid beetles in mountain ecosystems along with the consequences of possible reductions in sampling periods. Our results show that an early activity peak among carabids is predominant in mountain ecosystems. However, there are differences among species: the main group of species showed the highest activity directly after snow melt, a second group showed a delayed activity peak and a small third group had no clear peak at all. Based on this study, we recommend two fortnightly sampling periods as a minimum for a sampling programme: one immediately after snow melt, and a second sampling period after a pause of two weeks.

## Introduction

Since harsh abiotic conditions along with high spatial heterogeneity dominate mountain ecosystems, in stark contrast to the surrounding landscapes, alpine sites are interesting for ecological and biogeographical research (Lomolino 2001). This is especially true for questions related to environmental changes (e.g. climate change or change of land use) with mountains serving as suitable model ecosystems (Haslett 1997).

Carabidae is a group often used as indicator and/or model taxon (Dufrene and Legendre 1997; Rainio and Niemela 2003; Szyszko et al. 2000). They have also been used to help understand fundamental ecological processes in mountain landscapes (e.g. Franz 1970; Holdhaus 1954).

Nevertheless, very few investigations have been carried out using pitfall traps in high elevation mountain areas (cf. Brandmayr et al. 2003a; Gesellschaft für Angewandte Carabidologie 2009), although this method is otherwise very widely used. The most cited reason given for the limited use of this method is that of labour intensity due to the difficulty in accessing these often remote study areas. To reduce the time invested, depending on the research issue, it might be possible to shorten the sampling period. A likely side effect will be a lower number of specimens captured, leading most probably to a reduction in the number of recorded species. An understanding of the relationship between reduced sampling effort and number of recorded species is needed as a basis for decisions regarding timing and frequency of sampling, especially in the context of long-term monitoring.

Annual rhythms of activity and reproduction have been a major issue in carabidology, dating back to Larsson’s pioneering work in this field (Larsson 1939). His classification of carabids’ annual rhythms was elaborated upon and modified by other carabidologists (Lindroth 1949; Thiele 1977), and the importance of reproductive behaviour as a life history trait in carabids was emphasized by den Boer and van Dijk (1998) and Paarmann (1979). Many studies have dealt with the relationship between activity patterns and habitat preference, and these studies have lead to a better understanding of distribution trends and specific habitat adaptations of carabids (Lys and Nentwig 1991; Riddick and Mills 1995; den Boer and van Dijk 1996; Matalin 1997; Fadl and Purvis 1998; Traugott 1998; Hutchison 2007).

For mountain ecosystems, literature covering the reproductive seasonality of ground beetles is sparse. There are some works dealing with the phenology of carabids at high altitudes (Lang 1975; De Zordo 1979a, b; Janetschek et al. 1987; Gereben 1995; Ottesen 1996; Sota 1996; Hosoda 1999; Sharova and Khobrakova 2005). Many of these report a shortened activity period, but none of them examine possibilities and consequences of reduced sampling time.

A long-term research project in the “Allgäuer Hochalpen” in the German Alps was conducted in an area protected under the European Union ”Habitats Directive”. The project aimed at assessing effects of intensive long-term grazing of sheep and associated grazing regime changes after extensive cattle pasturing in 2000. In this project, intensive sampling of epigeic arthropods was performed over 6 years using pitfall traps. In this paper we focus on the seasonal activity of carabid beetles, primarily in the subalpine, but also in the alpine research area. We (1) describe the phenology of carabid beetles in the mountain ecosystems; and (2) investigate the consequences of reducing the number of sampling periods in order to present an optimized sampling scheme for recording the maximum number of species in subalpine and alpine altitudes of the northern Alps.

## Methods

### Study area

**Figure 1.**
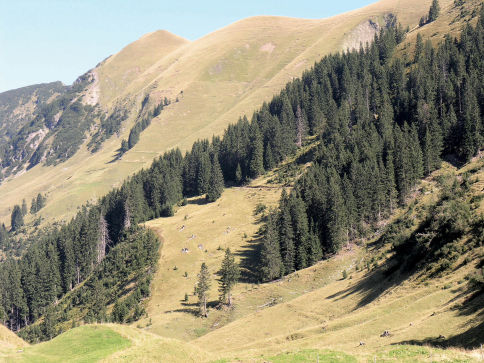
The study area “Alpe Einödsberg”. Position of some sampling sites is indicated.

The mountain pasture “Alpe Einödsberg“ (10,28°; 47,32°) is located in the German Alps (south-western Bavaria) and is part of the “Allgäuer Hochalpen”, an area protected under the European Union ”Habitats Directive”. The study area ranges in altitude from 1400 to 2000 meters above sea level (m a. s. l). and encompasses a total area of about 2 km². Most of the predominantly west-facing slopes consist of meadows dominated by *Nardus stricta*. Woodland belts dominated by Norway spruce (*Picea abies*) and krummholz made up of *Alnus viridis*, occur throughout the pasture zone (Fig. 1). There is a 2 km ridge running north-south along the upper segment of the pasture. Additional information about the vegetation and geology is given in Höfer et al. (2008), Höfer et al. (in press) and SMNK 2009.

In 2005, sampling was conducted at 25 sites. Sampling was focused on *Nardus stricta*-dominated meadows on slopes and on ridge sites dominated by *Deschampsia cespitosa*. In addition, several forest sites and open sites at lower altitudes were sampled (Table 1).

**Table 1.** Sample sites. Altitude is given in m a. s. l., incline and exposition in °.

*site*	*type*	*altitude*	*inclination*	*exposition*
V02	ridge	1875	12	180
V03	ridge	1880	21	210
V05	ridge	1885	29	275
V06	slope	1751	34	255
V08	slope	1776	35	260
V10	slope	1809	38	235
V11	slope	1703	27	250
V16	surface erosion	1790	35	230
V23	sucession *Alnus viridis*	1765	38	300
X01	ridge	1884	25	250
X03	slope	1896	33	270
X04	ridge	1980	32	280
X05	ridge	1993	9	250
X07	slope	1781	39	265
X08	slope	1786	35	260
X09	slope	1798	37	255
X10	ridge	1911	28	275
X11	slope	1751	34	300
X13	*Alnus viridis* krummholz	1750	38	320
X14	forest	1565	24	270
X15	forest	1550	34	285
X17	open, low altitude	1434	24	245
X18	open, low altitude	1476	31	270
X20	slope	1720	31	300
X21	ridge	1990	5	280

### Sampling

At each sampling site, 6 pitfall traps (with a diameter of 6 cm, filled with 10% acetic acid, 90% water) were installed at a distance of 6 m from each other. In order to protect the traps from heavy rain and from cattle-related damage, traps were placed in a metal tube with a transparent plastic cover (Lederbogen et al. 2004).

The pitfall traps were installed at the beginning of June, just after the first snowmelt at the ridge, and were removed at the end of September 2005 after a period of snow cover. Traps were emptied every fortnight. Altogether, there were 8 sampling periods, these were numbered chronologically (1: June 5th – 18th, 2: June 19th – July 2nd, 3: July 3rd – July 18th, 4: July 19th – August 1st, 5: August 2nd – August 15th, 6: August 16th – August 29th, 7: August 30th – September 12th, 8: September 13th – September 26th).

Carabids were identified to species level; the nomenclature of the species follows Müller-Motzfeld et al. (2004). Not all specimens of *Bembidion incognitum* and *Bembidion deletum* could be identified to species level and they were thus treated as ‘*Bembidion incognitum/deletum*’ in Table 2.

**Table 2.** List of carabids trapped over the whole sampling period and their traits ‘hindwing development’ (b: brachypter, d: dimorphic, m: macropteric) and body size class. For each species the sum of individuals caught (sum) as well as the percentage of individuals per sampling period 1 to 8 are given.

*Species*	*wing type*	*body size*	*sum*	*1 5.6.-18.6.*	*2 19.6.-3.7.*	*3 4.7.-18.7.*	*4 19.7.-1.8.*	*5 2.8.-15.8.*	*6 16.8.-29.8.*	*7 30.8.-12.9.*	*8 13.9.-26.9.*
*Pterostichus jurinei* (Panzer, 1803)	b	4	4431	30.1	33.2	16.1	7.3	2.7	1.9	4.7	4.0
*Pterostichus burmeisteri* Heer, 1838	b	5	2094	25.2	13.4	12.1	9.5	4.5	5.5	17.7	12.0
*Pterostichus unctulatus* (Duftschmid, 1812)	b	3	1386	24.4	21.2	17.0	11.8	5.3	8.0	9.2	3.2
*Pterostichus pumilio* (Dejean, 1828)	b	2	1044	20.3	15.8	23.7	23.6	8.7	2.6	2.7	2.7
*Pterostichus multipunctatus* (Dejean, 1828)	b	4	669	37.1	35.1	4.5	5.8	4.3	3.9	6.1	3.1
*Pterostichus melanarius* (Illiger, 1798)	d	5	578	13.5	31.1	30.3	11.8	5.4	3.1	4.0	0.9
*Abax parallelepipedus* (Piller & Mitterpacher, 1783)	b	5	577	9.5	25.1	16.8	19.6	7.3	10.1	10.6	1.0
*Trechus obtusus* Erichson, 1837	d	2	509	14.3	14.5	20.8	27.3	9.4	5.7	3.9	3.9
*Carabus auronitens* Fabricius, 1792	b	6	432	19.9	29.9	24.5	16.4	4.4	3.7	1.2	
*Carabus violaceus* Linné, 1758	b	7	365	5.5	21.9	27.4	29.0	6.8	6.6	2.5	0.3
*Amara erratica* (Duftschmid, 1812)	m	3	282	31.6	52.8	13.5	1.4	0.7			
*Leistus nitidus* (Duftschmid, 1812)	d	3	167	10.2	26.3	24.6	16.8	5.4	6.6	9.0	1.2
*Oreonebria picea* (Dejean, 1826)	b	4	159	32.7	30.8	16.4	15.1	2.5	1.9	0.6	
*Calathus melanocephalus* (Linné, 1758)	d	3	102	5.9	2.9	28.4	17.6	26.5	12.7	4.9	1.0
*Pterostichus strenuus* (Panzer, 1796)	d	3	97	52.6	23.7	11.3	6.2		2.1	1.0	3.1
*Calathus micropterus* (Duftschmid, 1812)	b	3	76	14.5	23.7	28.9	14.5	3.9		11.8	2.6
*Harpalus latus* (Linné, 1758)	m	4	74	10.8	20.3	12.2	21.6	16.2	12.2	6.8	
*Poecilus versicolor* (Sturm, 1824)	m	4	73	26.0	52.1	12.3	4.1	4.1		1.4	
*Pterostichus diligens* (Sturm, 1824)	d	2	73	57.5	20.5	11.0	4.1			2.7	4.1
*Dyschirius globosus* (Herbst, 1784)	d	1	51	54.9	7.8	15.7	13.7	3.9	2.0		2.0
*Cicindela campestris* Linné, 1758	m	4	44	15.9	22.7	27.3	13.6	11.4	2.3	4.5	2.3
*Carabus sylvestris* Panzer, 1796	b	6	36		58.3	16.7	2.8	8.3	2.8	8.3	2.8
*Amara aulica* (Panzer, 1797)	m	5	32		6.3	50.0	43.8				
*Bembidion bipunctatum nivale* Heer, 1837	m	2	32	84.4	3.1	9.4				3.1	
*Trichotichnus laevicollis* (Duftschmid, 1812)	d	3	30	26.7	23.3	13.3	23.3	3.3	6.7	3.3	
*Amara lunicollis* Schiödte, 1837	m	3	24	29.2	62.5	4.2	4.2				
*Notiophilus biguttatus* (Fabricius, 1779)	d	2	23	8.7	17.4	17.4	30.4	4.3	4.3	13.0	4.3
*Bembidion incognitum/deletum*	m	2	19	52.6	21.1	15.8		10.5			
*Cychrus attenuatus* (Fabricius, 1792)	b	5	19	5.3	5.3	15.8	36.8		5.3	21.1	10.5
*Amara nigricornis* C.G. Thomson, 1857	m	3	17	17.6	41.2	17.6	17.6			5.9	
*Nebria rufescens* (Stroem, 1768)	m	4	17	23.5	23.5	23.5	11.8	11.8		5.9	
*Bembidion lampros* (Herbst, 1784)	d	2	15	73.3	6.7	13.3					6.7
*Cychrus caraboides* (Linné, 1758)	b	5	11	27.3	45.5	9.1		9.1	9.1		
*Bembidion properans* (Stephens, 1828)	d	2	7	14.3	57.1	28.6					
*Loricera pilicornis* (Fabricius, 1775)	m	3	6	16.7	66.7	16.7					
*Nebria brevicollis* (Fabricius, 1792)	m	4	3	33.3		33.3	33.3				
*Agonum sexpunctatum* (Linné, 1758)	m	3	2			100.0					
*Acupalpus flavicollis* (Sturm, 1825)	m	2	1		100.0						
*Amara familiaris* (Duftschmid, 1812)	m	3	1			100.0					
*Amara praetermissa* (C.R. Sahlberg, 1827)	m	3	1					100.0			
*Carabus glabratus* Paykull, 1790	b	7	1			100.0					
*Chlaenius nigricornis* (Fabricius, 1787)	m	4	1	100.0							
*Cicindela sylvicola* Dejean, 1822	m	5	1			100.0					
*Harpalus affinis* (Schrank, 1781)	m	4	1						100.0		
*Pterostichus vernalis (*Panzer, 1796)	d	3	1			100.0					
*Synuchus vivalis* (Illiger, 1798)	d	3	1					100.0			
*Total*	* *	* *	*13585*	*24.9*	*25.8*	*17.2*	*12.1*	*4.8*	*4.1*	*7.0*	*4.2*

### Data analysis

In order to compare species and sites with different numbers of individuals, percentage of total catches per sampling period were used. Total number of species per sampling period and mean number of species per site and sampling period were compared.

For comparison of phenology at different altitudes, the sites were divided into three altitude classes (<1600, 1600–1850, >1850 m a. s. l.) which contained similar numbers of sites. The weighted mean phenological appearance was calculated for each species per altitude class. Differences among classes were tested with a t-test, whereby we only used data for species which occurred at each sampling site and for which at least 10 individuals per class were found. Differences in phenological appearance in the traits: ‘hindwing development’ and ‘body length’ were also tested. For ‘hindwing length’ the groups ‘brachypter’, ‘dimorphic’ and ‘macropterous’ were tested using a t-test; for body size, species were grouped into 7 classes (mean body length < 3 mm, 3–6 mm, 6–9 mm, 9–12.5 mm, 12.5–20 mm, 20–27 mm, >27 mm) and a Spearman rank correlation was performed. Bonferroni corrections were conducted for each test family. For t-tests, data were checked for normality with Shapiro-tests.

Species accumulation curves (also called sample-based rarefaction curves) were used to compare sampling effort and species richness measures (Buddle et al. 2005; Duelli et al. 1999; Gotelli and Colwell 2001; Ugland et al. 2003). Rarefaction curves for the whole dataset and for each unique sampling period were calculated using Kobayashi’s formula (Kobayashi 1974) in the R package ‘vegan’ (Oksanen et al. 2008; R Development Core Team 2008).

Several reduced datasets with data from two sampling periods were produced. Rarefaction curves and species number per site were compared across the whole dataset, single sampling periods and different combinations of sampling periods.

To understand how the assemblage of carabid beetle species could be represented in the case of a reduced sampling effort we computed a dissimilarity matrix based on Bray-Curtis distances. For this analysis, species numbers were standardised to percentage-data of total species numbers per site and square-root-transformed. Based on this matrix a hierarchical cluster procedure was conducted using Ward´s minimum variance method.

## Results

In total, 13,585 specimens representing 47 species of carabid beetles were trapped over the sampling period (Table 2). The 10 most abundant species occurred throughout the whole altitudinal range of the study area.

General seasonal activity began with a maximum at the beginning of the study and showed a continuously decreasing tendency up until the end of September (Fig. 1). Focusing on individual numbers, the highest activity was in June, where >50% of individuals were caught. In July, activity declined slowly, and in the second half of the sampling timespan, August and September combined, only 20% of the total number of individuals were trapped.

**Figure 2.**
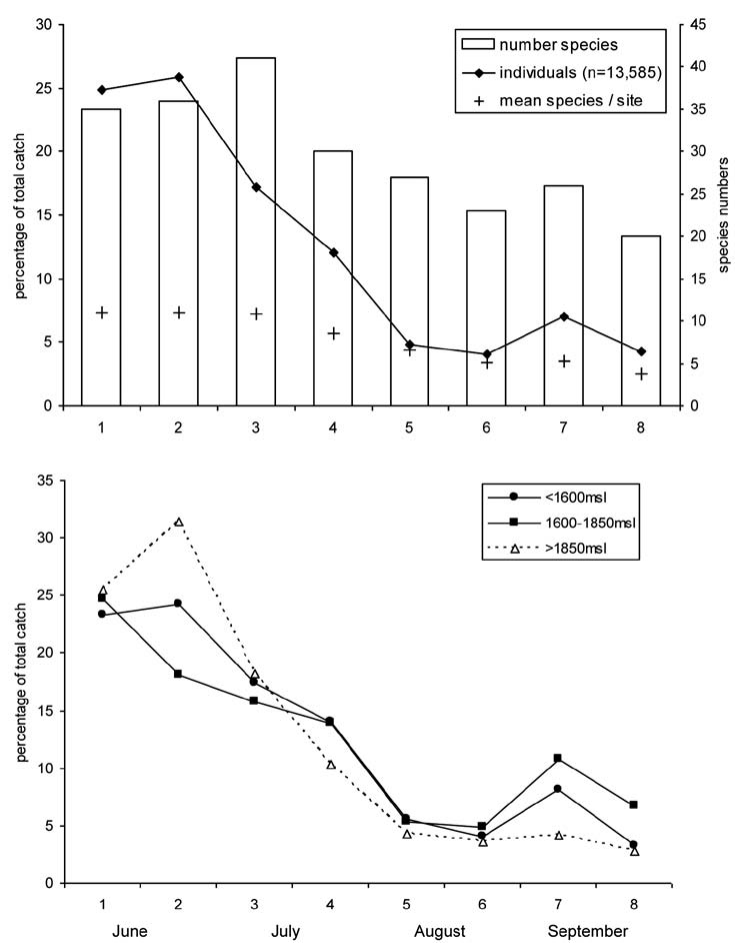
Phenology of ground beetles. **a** Overview over all sites. Number of individuals is converted to percentage of total catch. **b** Seperated for the three site classes of altitude. On the horizontal axis the sampling interval is given. For exact sampling periods, see Table 2.

The number of recorded species follows a similar trend: after a minute increase up to the first half of July, the number of species decreased. Mean species number per site was almost constant from June to the beginning of July, and then decreased up to the end of the study period (Fig. 2a).

The extremes in beetle activity were greatest at sites above 1850 m a.s.l. relative to the other two altitude classes: the maximum in June was higher and the low activity from the second half of July until September was even more pronounced (Fig. 2b). Differences between the activity phenology of low and mid-altitude classes were not significant (t=-0.322, p=0.753); however, mean activity of ground beetle species occurring at all elevations was earlier at higher altitudes than at the mid-altitude and lower sites (t=4.33, p=0.001).

All species of which at least 10 individuals were caught had their activity peak in June or July, approximately two thirds of the species in June, and one third in July (Table 2).

**Figure 3.**
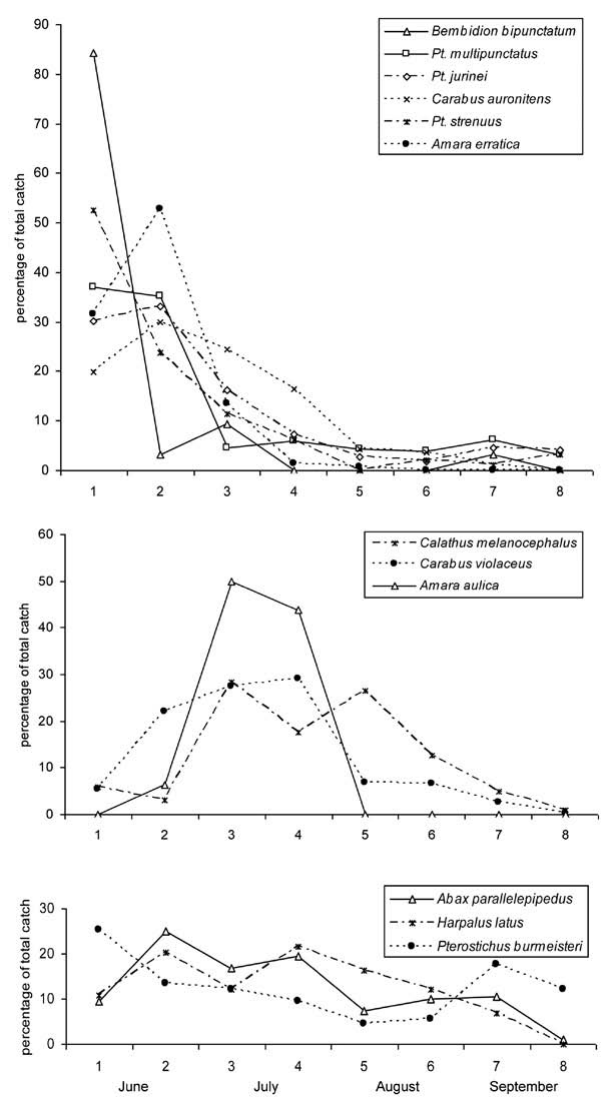
Phenology of single species. **a** Species with an early activity peak, **b** Species with a delayed activity peak and **c** Species without a clear activity peak. On the horizontal axis the sampling interval is given. For exact sampling periods, see Table 2.

Species can be divided into three groups according to their phenology: (1) The first and largest group of species shows quite a distinct activity peak in June (Fig. 3a) and often a strong decline already occurring in July (e.g. *Pterostichus multipunctatus*, *Bembidion bipunctatum*). Some of these species are almost absent in the second half of the year (*Amara erratica*, *Bembidion bipunctatum*). The strength of the spring activity peak may also be less pronounced (e.g. *Carabus auronitens*). (2) A second group of species shows a delayed activity peak (Fig. 3b). In most cases, the magnitude of the peak was weaker than seen in the early species. In species with a delayed activity peak, there are also cases with absence in the second half of the year (*Amara aulica*). (3) The third group is comprised of species that show no clear activity peak (Fig. 3c), i.e. which are active over the entire sampling timespan. Only a few species fit into this latter scheme. Most of these are characterized by a weak peak in June, followed by a slow decline in activity. Some of the species exhibit a relatively high activity in the autumn (*Pterostichus burmeisteri*, *Abax paralellepipedus*).

We were unable to find any significant relationship between phenological appearance and hindwing development of the species. Similarly, for body size no significant difference was found, although there is a weak trend of larger species appearing later in the year (t=1.61, df=44, p=0.114).

**Figure 4.**
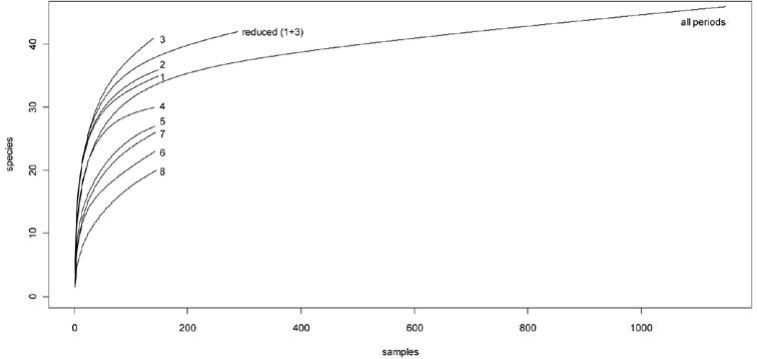
Sample-based rarefaction curves. Numbers refer to the different sampling periods.

The sample-based accumulation curves of the first three sampling periods are almost identical at the start, and considerably steeper than the curves of subsequent periods and the curve based on the entire dataset (Fig. 4). After 25 samples, the curves of the first three periods split: the third period curve attains higher values, and the first and second period curves follow a similar trend. Decreasing overall activity after the spring peak is also discernible in the rarefaction curves: the curves for sampling periods 4–8 are clearly below the curve for complete sampling (the curve for sampling period 4 is greater than the total sample curve at the start of the rarefaction process and then falls below it).

Table 3 illustrates the effects of a reduced sampling effort on observed species richness. While single sampling periods achieve a maximum of 65% of the total number of species over the entire sampling timespan, a combination of two early sampling periods can exceed 80% of the total amount of species (sampling periods 1 and 2 or 1 and 3). Best results are obtained when sampling effort is reduced to sampling periods 1 and 3. With the reduced datasets for sampling periods 1 and 3, 91.3% of all species were detected. Per site, the mean quota was 83%, and varied between 68.8 and 100%. The quota of species detected was seen to be independent of altitude or number of specimens caught.

**Table 3.** Comparison of species trapped per site for different sampling efforts. In the column Species the total number of species per site is given. Percentage of species caught is indicated for each single sampling period and a selection of two combined periods. The mean percentage of species caught per site (mean/site) for each effort is calculated.

*site*	*single periods*								*two periods*				*species *
	*1 5.6.-18.6.*	*2 19.6.-3.7.*	*3 4.7.-18.7.*	*4 19.7.-1.8.*	*5 2.8.-15.8.*	*6 16.8.-29.8.*	*7 30.8.-12.9.*	*8 13.9.-26.9.*	*1+2*	*1+3*	*2+3*	*1+4*	
V02	64	59	73	41	23	23	32	14	86	86	82	73	22
V03	68	64	73	32	36	32	32	27	86	86	82	73	22
V05	76	76	57	57	19	14	19	10	90	86	90	81	21
V06	56	75	50	38	56	44	38	6	81	69	75	63	16
V08	77	54	69	62	62	62	31	15	77	92	77	77	13
V10	45	65	80	60	45	40	25	20	65	85	95	65	20
V11	63	69	69	50	44	13	31	25	81	88	81	75	16
V16	71	53	47	29	41	29	18	12	71	88	71	76	17
V23	53	100	60	67	47	27	33	7	100	73	100	80	15
X01	82	88	71	47	24	12	18	24	94	88	94	82	17
X03	64	57	71	43	50	36	36	29	86	86	86	64	14
X04	61	56	50	44	44	44	33	28	72	72	67	72	18
X05	73	68	77	64	45	36	36	32	95	100	82	91	22
X07	53	53	60	73	60	40	20	27	67	73	67	80	15
X08	73	60	60	60	47	47	47	27	87	87	73	80	15
X09	83	67	67	67	50	33	33	25	92	83	83	100	12
X10	46	92	77	38	46	38	23	15	92	85	100	54	13
X11	63	53	63	42	37	37	32	32	68	79	68	68	19
X13	65	59	65	47	18	18	53	35	71	88	82	76	17
X14	53	73	67	53	33	47	47	33	87	80	80	60	15
X15	71	57	71	93	50	43	57	29	79	86	71	93	14
X17	79	43	50	43	36	14	21	21	79	79	50	86	14
X18	53	37	58	32	21	11	26	11	63	79	63	63	19
X20	63	69	44	56	44	25	19	44	81	69	88	69	16
X21	68	73	68	50	27	18	27	14	82	86	86	73	22
all sites	76	78	89	65	59	50	57	43	83	91	91	80	46
mean/site	65.4	65.3	64.8	52	40.9	32	32.4	23.2	81.3	82.9	79.7	75	100

**Figure 5.**
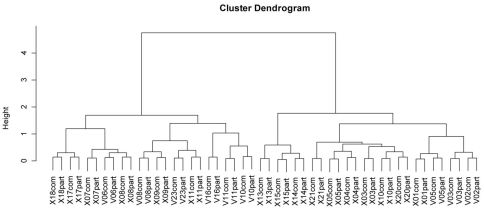
Dendrogram of sites with data from complete sampling (comp) and sampling periods 1 and 3 (part). The dendrogram is based on Bray-Curtis distances and uses Ward´s minimum variance method.

Classification showed that a reduced dataset (periods 1 and 3 only) represents the assemblage structure in a similar way to the complete dataset: All sites are grouped together until the last splitting, where they are divided as a result of sampling intensity (Fig. 5).

## Discussion

The strong activity peak observed in our study at the start of the plant growing season has already been observed in many carabids in mountain regions, especially in subalpine and alpine ecosystems (Lang 1975; De Zordo 1979a, b; Refseth 1984; Janetschek, Meyer, Schatz and Schatz-de Zordo 1987; Gereben 1995; Ottesen 1996; Brandmayr et al. 2003b; Löffler and Finch 2005; Sharova and Khobrakova 2005). Depending on altitude, exposition and longitude, the weeks immediately after snow-melt are characterised by an activity peak in many species. The shortened plant growing season and the time in which the larvae are able to develop are given as an explanation for this. A fast start to reproduction in cool ecosystems is advantageous, as larval development takes longer under such conditions (cf. Paarmann 1966; Ferenz 1975). Food availability is another possible explanation; many swarming or flying insects (especially Diptera and Hymenoptera) are attracted to the white snow fields. After landing on these fields the insects are immobilised by the low temperatures. In most cases, ground beetles can pick them up live overnight or, later, as carcasses on snow fields after snowmelt. In these cases, some authors use the term “snow edge species”, i.e. species which are adapted to cold and humid conditions and disappear very rapidly after snowmelt (Holdhaus 1954; Franz 1970; Marggi 1992; Brandmayr et al. 2005).

However, snow edge species (Marggi 1992), such as *Bembidion bipunctatum nivale*, are not the only species most often trapped after snow melt. Species with broader habitat preferences show increased activity during that time. Prevailing conditions after snow melt (open structure of vegetation and low “Raumwiderstand” sensu Heydemann 1956, the resistance of vegetation structure to the locomotory movement of a given species, high temperatures during sunshine combined with a high soil humidity) might be advantageous for several species.

While most papers focus on the abundant species, our data show that an early activity peak can also be observed for less abundant species. Ottesen (1996) made the same observation for carabids in alpine sites in Norway. However, this was not true for other groups of epigeic arthropods, as he observed an autumn activity peak for some species of staphylinids.

Although high spring activity was seen for all species, we observed differences between species: we were able to divide our species into three groups according to their phenological appearance. While the first group (early and strong spring activity peak) is most frequently described in mountain ecosystems, delayed spring activity has already been shown for some species by other authors (De Zordo 1979b; Refseth 1984), and a species without a strong activity peak was presented (Sharova and Khobrakova 2005). Our data do not give a clear indication of the reasons for the observed phenological differences among species, at least there were no simple relationships between the traits we tested.

The observed activity densities of species, with some species having a strong and early peak and others with a delayed peak led us to the conclusion that the best results can be expected by reducing the sampling to two periods at the beginning of the season. In fact, sampling periods 1 and 3 showed the highest average coverage of the sites’ species richness, and the quota of species trapped was better than that suggested by Duelli et al. (1999) for the so-called standard minimum programme for lower altitudes. This means that for high altitudes our suggestion to reduce the sampling effort results in a more robust data set than similar approaches for lower altitudes, as the beetles’ activity seems to be more concentrated within a shorter period in mountain ecosystems. The phenological data of other analyses conducted in different habitats from the upper montane zone upwards supports an approach that focuses on an early first sampling period and a delayed second (Lang 1975; De Zordo 1979a, b; Refseth 1984; Janetschek, Meyer, Schatz and Schatz-de Zordo 1987; Gereben 1995; Ottesen 1996; Löffler and Finch 2005; Sharova and Khobrakova 2005). Results of the classification procedure showed that with our reduction in sampling effort, community structure is represented well. A reasonably reduced sampling effort improves the chances of including carabid beetles in monitoring programmes in mountain areas, e.g. to evaluate the conservation status of habitats in Natura 2000 areas. If a reduction in sampling effort is inevitable, we recommend that the minimum sampling effort for carabids in mountain ecosystems should be two fortnightly sampling periods, the first immediately after snow melt and a second after a break of two weeks.
